# Changes of the cardiac electrical cycle in type 1 diabetes during hypoglycaemia – a prospective observational study

**DOI:** 10.3389/fendo.2025.1690371

**Published:** 2026-01-06

**Authors:** Árpád Kézdi, Viktor J. Horváth, Viktor Vass, Márk M. Svébis, Győző Kocsis, Viktória Ferencz, Tamás Jávorfi, Beatrix A. Domján, Ádám G. Tabák

**Affiliations:** 1 Department of Internal Medicine and Oncology, Semmelweis University, Budapest, Hungary; 2 Rácz Károly Conservative Medicine Division, Doctoral College, Semmelweis University, Budapest, Hungary; 3 Institute of Preventive Medicine and Public Health, Semmelweis University Faculty of Medicine, Budapest, Hungary; 4 Diabetes Outpatient Clinic, Jahn Ferenc South Pest Hospital and Clinic, Budapest, Hungary; 5 UCL Brain Sciences, University College London, London, United Kingdom

**Keywords:** cardiac arrhythmias, continuous glucose monitoring, “dead-in-bed” syndrome, hypoglycaemia, QTc interval, type 1 diabetes

## Abstract

**Introduction:**

The “dead-in-bed” syndrome is thought to be a consequence of hypoglycaemia-induced QTc prolongation in type 1 diabetes. Thus, we characterized electrocardiogram (ECG) changes during hypoglycaemia in healthy, free-living patients with type 1 diabetes without major comorbidities.

**Methods:**

A cohort of n=23 patients with type 1 diabetes wore continuous subcutaneous glucose and ECG monitors for approximately 7 days. We compared the frequency of ventricular premature beats (VPBs), the mean and SD of heart rate, and ECG intervals during hypoglycaemic episodes and their respective control episodes (n=226 episodes; n=1,697,205 beats) using linear mixed models.

**Results:**

The mean duration of hypoglycaemic episodes was 159 min (95% CI: 128**–**192) at night and 66 min (95% CI: 57**–**75) during the day. No differences in any of the investigated parameters were found between hypoglycaemic and control episodes during the day, nor in the frequency of nighttime VPBs. In contrast, the mean corrected QT (QTc) interval (mean difference (MD): 5, 95% CI: 2–9 ms) and the SDs of RR (MD: 17, 95% CI: 4–31), P-wave (MD: 2, 95% CI: 0–4), PQ (MD: 3, 95% CI: 1–6), QT (MD: 4, 95% CI: 2–7), and QTc intervals (MD: 5, 95% CI: 2–8) increased during nighttime hypoglycaemia.

**Discussion:**

Daytime hypoglycaemia is an unlikely cause of malignant arrhythmias in healthy patients with type 1 diabetes. The minimal increase in the QTc interval alone does not suggest an increased risk of malignant arrhythmias. However, the increase in the mean QTc together with an increase in its SD during nighttime hypoglycaemia is compatible with the extremely rare occurrence of the “dead-in-bed” syndrome.

## Introduction

Cardiovascular disease (CVD) is the leading cause of morbidity and mortality in patients with diabetes, with long-term glycaemic control being a major determinant of vascular complications (1, 2). Given that, in parallel with improving glycaemic control, the risk of hypoglycaemia is increasing in patients with type 1 diabetes, hypoglycaemia remains the major limiting factor of glycaemic management (3). Starting with the publication of a case series of 22 young patients with type 1 diabetes who suffered an overnight death and in whom autopsy revealed no abnormalities, further reports described sudden cardiac death likely caused by hypoglycaemia (“dead-in-bed” syndrome) (4–7).

Although several theories exist, the exact pathomechanism of the “dead-in-bed” phenomenon has not yet been elucidated. While nighttime hypoglycaemia is an everyday problem in type 1 diabetes, “dead-in-bed” syndrome is extremely rare, suggesting that an unfortunate combination of factors could trigger its development (8).

Hypoglycaemia can lead to a proarrhythmic state through several mechanisms, including disturbances of intracellular and serum potassium and calcium metabolism, as well as increased β-adrenergic sympathetic tone. Furthermore, microvascular and macrovascular diabetes complications are also associated with an increased risk of arrhythmias. All these mechanisms can lead to a prolonged QT interval, increased heart rate (HR), and a more frequent occurrence of cardiac ectopias (9).

This is well supported by hypoglycaemic clamp studies that report substantial mean increases in the QTc interval (ranging from 30 to 230 ms) (10–14). In contrast, the increase in QTc interval is an order of magnitude smaller in free-living patients (ranging from 3 to 30 ms, mostly 5–10 ms) (15–20) which is unlikely to trigger dangerous arrhythmias. However, these studies only describe mean changes in the QTc interval, and variability of the QTc intervals and the extremes of QTc values have not yet been investigated—although even a single long-QT beat may initiate a life-threatening arrhythmia (21, 22).

To better characterise electrocardiogram (ECG) changes during hypoglycaemia and potential underlying causes of the “dead-in-bed” syndrome, we performed simultaneous continuous subcutaneous glucose (CGM) and ECG monitoring in free-living patients with type 1 diabetes for approximately 7 days. Using a case–control design, we then compared the differences in: (1) the means and (2) the standard deviations (SDs) of each episode of the cardiac cycle, as well as (3) HR and (4) the frequency of ventricular ectopias between normoglycaemic and hypoglycaemic episodes.

## Materials and methods

### Setting

We report the results of an observational study conducted at the Department of Internal Medicine and Oncology, Semmelweis University (Budapest, Hungary), between 19/JAN/2015 and 2/FEB/2016. First, participants were checked for eligibility. After baseline assessment, all included patients underwent simultaneous 12-lead Holter ECG and single-blinded CGM monitoring for up to 168 hours while continuing their usual daily activities and diabetes management.

The study was conducted in accordance with the Declaration of Helsinki. Oral and written informed consent was obtained from all participants prior to inclusion in the study. The study protocol was approved by the National Scientific and Ethics Committee of the Hungarian Medical Research Council (ETT TUKEB 507/2014).

### Selection of participants and observations

Altogether, 31 individuals with insulin-treated type 1 diabetes, aged 18–55 years and with a diabetes duration of ≥5 years, were included. Exclusion criteria were treatment with antiarrhythmic drugs (e.g., amiodarone, sotalol) other than beta-blockers; ECG abnormalities that could hinder differentiation of supraventricular and ventricular premature beats (e.g., complete bundle branch block, Wolff–Parkinson–White (WPW) syndrome); non-sinus rhythm (e.g., high-degree atrioventricular block, atrial fibrillation); known macrovascular diabetes complications (acute myocardial infarction (AMI), stroke, transient ischemic attack, or peripheral arterial disease); chronic kidney disease stages 3 and 4 (23); hypo- or hyperthyroidism unless thyroid-stimulating hormone (TSH) was within the normal range.

**Figure 1:**
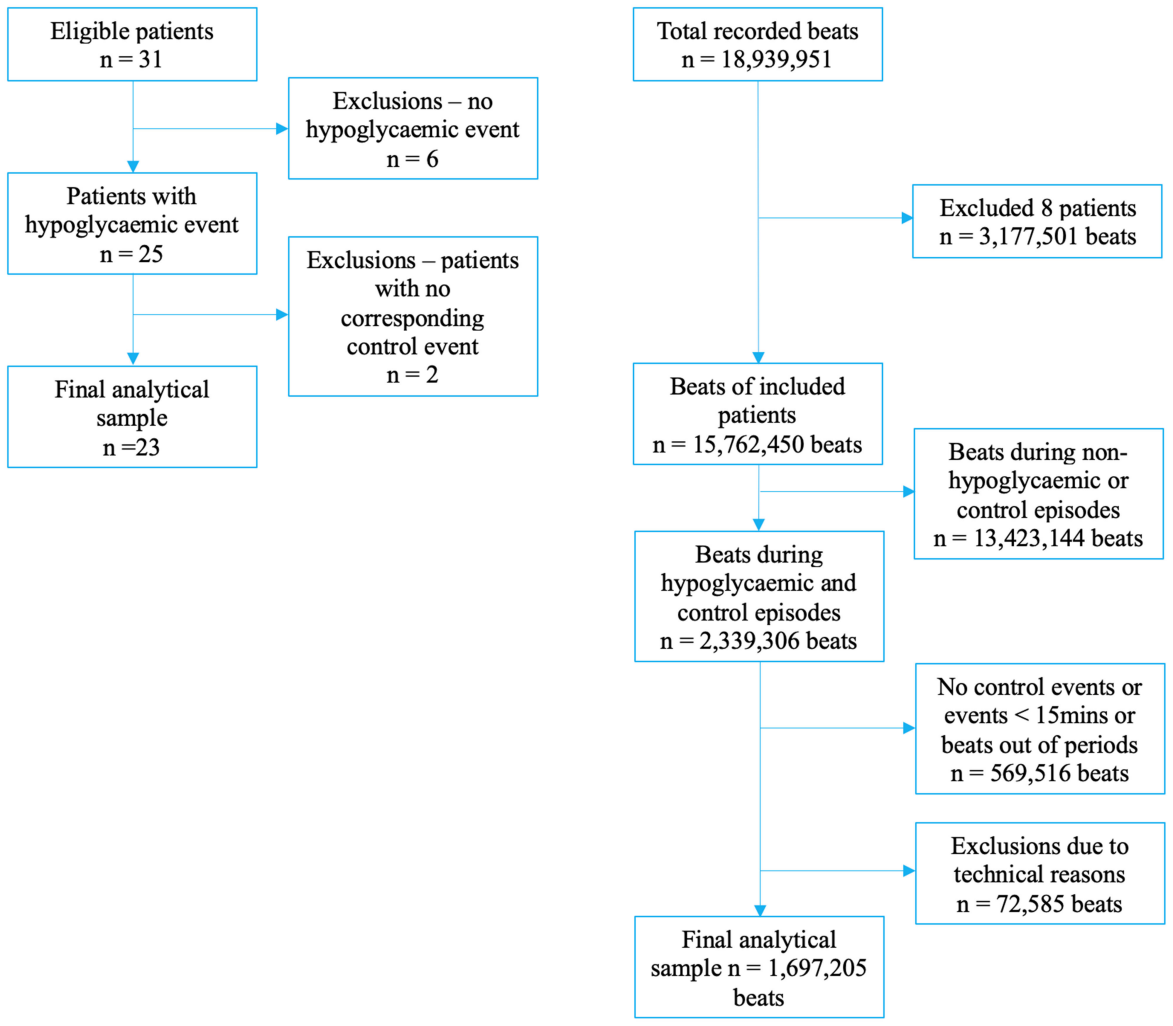
Flow chart of participants’ selection.

Of the n=31 included patients, we excluded n=6 (19%) due to the lack of hypoglycaemic episodes during monitoring. A further n=2 (6%) were excluded due to the lack of adequate control episodes. The final analytical sample comprised n=23 (74%) patients (**Figure 1**).

The second level of exclusion was applied at the level of beats. Of the original 18,939,951 beats (n=31 patients), we excluded 3,177,501 beats (16.8%; n=8 patients). The remaining 23 patients had a total of 15,762,450 beats. Given that ours is a case–control analysis, we included only beats from hypoglycaemic episodes and their respective control episodes (n=2,339,306 beats) (**Figure 1**).

Of these eligible beats, we excluded those recorded during hypoglycaemic episodes lasting <15 min or without an adequate control episode (569,516 beats, 24.4%). Finally, we excluded individual beats in which the measured ECG parameters were clearly erroneous or improbable (clinically too low or high), totalling 72,585 beats (3.1%). Thus, our final analytical sample included 1,697,205 beats (72.6% of those eligible) (**Figure 1**).

### Screening and baseline assessment

Before any further study-related procedures, participants were checked for eligibility. Data on age, medical history (type and duration of diabetes, macrovascular diabetes complications, etc.), and information on concomitant medications were collected from electronic health records (EHR) and medical examination. Laboratory tests and a baseline 12-lead ECG were performed (if not available in the EHR within 30 days) to check for exclusion criteria.

After eligibility screening, further data were collected at the baseline visit from EHRs and medical examinations, including a questionnaire: details of insulin treatment (insulin doses and delivery method), concurrent medications (beta-blockers, thyroid hormone replacement), smoking status, family history of AMI, anthropometric data (weight, height, BMI, waist and hip circumference), systolic and diastolic blood pressure, presence of sensory and autonomic neuropathy, and other laboratory results.

Smoking status was coded as never, ex-, or current smoker (≥1 cigarettes/day). Positive family history of AMI included all relatives.

Body weight was measured in light clothing without shoes using a calibrated digital scale (ADE M320000–01 electronic weight scale, ADE, Germany) and rounded to the nearest 0.1 kg. Height was measured without shoes and rounded to the nearest centimetre. BMI was calculated as weight (kg)/height^2^ (m). Waist circumference was measured at the midpoint between the iliac crest and the lowest point of the ribcage after a normal exhalation. Hip circumference was measured at the level of the greater trochanter. Both were rounded to the nearest centimetre (24).

Resting sitting blood pressure was measured three times using a calibrated digital meter (OMRON M4-I, Omron Electronics Kft., Budapest, Hungary) after a 5 min rest. The average of the three measurements was used.

Cardiovascular autonomic neuropathy was assessed using the tests proposed by Ewing et al., apart from the handgrip test. It was diagnosed if at least two tests were abnormal (25, 26). For the diagnosis of distal symmetric polyneuropathy, tuning fork and 10 g Semmes–Weinstein monofilament tests were used. It was diagnosed if symmetrical lower limb positivity was found (≥4 for monofilament or ≤6 for tuning fork).

Serum creatinine, urinary albumin-to-creatinine ratio, TSH, HbA1c, serum lipids (total, HDL, and LDL cholesterol), and N-terminal prohormone of brain natriuretic peptide were measured, and estimated glomerular filtration rate was calculated by the same central laboratory (Central Laboratory of Semmelweis University) on automated systems, not earlier than 30 days before baseline examination. The laboratory is accredited by the Hungarian Association for Clinical Chemistry.

### Glucose and ECG monitoring

All patients underwent a maximum 168-hour long simultaneous 12-lead Holter ECG (Labtech EC-12H device, Labtech Ltd., Budapest, Hungary) (27) and CGM monitoring (iPro2 CGM monitoring system with Enlite sensor, Medtronic Hungária KFT, Budapest, Hungary) (28), while continuing their usual daily activities and diabetes management.

Calibrations with self-monitored blood glucose values were performed at least four times daily according to the manufacturer’s recommendations during the study period. The sensor reports interstitial glucose (IG) values between 2.2 and 22.2 mmol/L every 5 min. This CGM system has been shown to be clinically accurate, with 97.3% of points within the A+B zones of the Clarke error grid when compared with fingerprick blood glucose values. The mean absolute difference between sensor and meter glucose values was approximately 14%, and hypoglycaemia detection rate was in the range of 80–90%. The lag time between meter glucose and sensor glucose was 8 ± 13 min, suggesting that a lag time >34 min was rare (29, 30).

### CGM predictors

For the purposes of the current analysis, we defined non-hypoglycaemia as an IG value >3.5 mmol/L and hypoglycaemia as an IG ≤3.5 mmol/L. The latter value lies between the generally accepted cut-offs for level 1 (3.9 mmol/L) and level 2 (3.0 mmol/L) hypoglycaemia (31, 32). We selected this cut-off because recent research suggests that counter-regulatory mechanisms—thought to be partly responsible for the cardiovascular effects of hypoglycaemia—are triggered at glucose levels between level 1 and level 2 hypoglycaemia (33).

A valid hypoglycaemic episode was defined as a period of hypoglycaemia lasting ≥15 min. The first IG reading ≤3.5 mmol/L marked the start of the episode, and the first IG reading >3.5 mmol/L marked its end. For each hypoglycaemic episode, we systematically checked for a control episode during the following or preceding days at the same time period. The first non-hypoglycaemic period was used as the control episode if no hypoglycaemia had been detected in the preceding 1 h. Due to technical reasons, the lengths of the hypoglycaemic and control episodes could differ; this was accounted for in the statistical analysis.

For a sensitivity analysis, a subset of hypoglycaemic episodes with the lowest IG values was defined as the nadir episodes. The lowest IG value within a given hypoglycaemic episode was designated as the hypoglycaemic nadir. Starting from the nadir, a 15 min nadir episode was constructed. As none of the hypoglycaemic episodes ended within 15 min of the nadir, no exclusions were required. The control episode for the nadir episode was selected using the procedure described above.

Another sensitivity analysis was conducted using the definition of the main analysis except for the glucose cut-off, which was set at 3.0 mmol/L.

All hypoglycaemic and control episodes were categorised into daytime (6 a.m. to 10 p.m.) and nighttime (10 p.m. to 6 a.m.) episodes.

### ECG outcomes

Continuous 24 h, 12-lead ECG recordings were obtained using a Labtech EC-12H digital Holter system and analysed with Cardiospy software (version 5.4) (34). Standard Mason–Likar electrode placement was applied for 12-lead configuration. Signals were recorded at a sampling frequency of 500 Hz with an analog band-pass filter of 0.05–150 Hz and a 50 Hz notch filter. Recordings were automatically processed using Cardiospy, which separates normal ECG traces from artefacts and detects arrhythmic events (such as ventricular premature beats (VPBs)) according to the manufacturer’s default event definitions. The automatic evaluation was manually reviewed by an experienced investigator (VJH). Artefacts, noise, and ectopic beats were visually inspected and corrected or excluded according to the standards of heart rate variability analysis (35, 36).

### Arrhythmia analysis

The definition of VPBs included both simple and complex VPBs (such as couplets and runs). The number of verified VPBs for each episode was used as the unit of analysis.

### Analysis of normal beats

For the analysis of normal beats, we selected only those detected as normal by Cardiospy and not excluded during expert review. The following intervals were considered: RR interval, P-wave length, PQ, QRS, QT, and QTc intervals. RR values were also expressed as HR (HR = 1000*60/RR (ms)).

As most previous reports on the effect of hypoglycaemia on QTc length were based on Bazett’s formula, we selected this method for the main analysis. For sensitivity analyses, we also calculated QTc using the Fridericia, Framingham, and Hodges corrections (15–17, 19, 20, 37, 38). We further determined the frequency of normal beats with QTc >500 ms (Bazett’s correction) for each episode as a sensitivity analysis, as this is generally accepted to be associated with an increased risk of malignant arrhythmia (39).

As previous observations suggested that diabetic autonomic and sensory neuropathies, as well as other microvascular complications, are associated with increased QTc duration (based on the Bazett correction), we ran a sensitivity analysis to investigate the potential interaction between neuropathy (defined as the presence or absence of any neuropathy) and hypoglycaemia status on QTc and its SD (40–42).

For each episode, the simple arithmetic mean and SD (as a measure of variability) of each interval (e.g., mean of P-wave lengths and SD of P-wave lengths) were used as outcomes in further analysis. The SD was selected based on the generally accepted hypothesis that arrhythmias are induced by beats with extreme characteristics, which may be better reflected by a measure of dispersity than by the mean (21, 22).

### Statistical analysis

No formal power calculations were performed before the study. Sufficient power was expected since previous studies with similar or smaller sample sizes detected significant increases in QTc interval (15, 18–20).

*Descriptive statistics* were provided for all baseline characteristics as mean ± SD for normally distributed variables, median (interquartile range [IQR]) for skewed continuous variables, and percent (%) for categorical variables. Differences between included and excluded participants were investigated using Mann–Whitney U tests and independent-samples t-tests for skewed and normally distributed continuous variables, respectively, or chi-square tests for categorical variables.

Given that each participant could provide more than one hypoglycaemic episode for analysis, differences between hypoglycaemic and control episodes, as well as between daytime and nighttime episodes, were analyzed using linear mixed models with a random effect for each participant and each event within participants, with an unstructured covariance matrix. Duration of episodes and lowest IG within each episode were used as outcomes, and hypoglycaemia status and time of day (daytime/nighttime) were included as fixed effects. The square root of episode durations was used due to skewness in the distribution. Data are presented separately for daytime and nighttime episodes.

For the *normal*-*beats analysis*, similar models were constructed with mean and SD of RR interval, P-wave length, PQ, QRS, QT, and QTc intervals as outcomes. As a sensitivity analysis, the normal-beats analysis was repeated for the nadir episodes and their respective control episodes.

Given that each participant could provide more than one hypoglycaemic episode, the comparison of the *frequency of VPBs* and of *beats with prolonged QTc* (>500 ms) between hypoglycaemic and control episodes during daytime and nighttime was performed using a generalised linear mixed model (negative binomial distribution with log link to control for overdispersion), with time of day (day or night) and episode type (hypoglycaemic or control) as fixed factors. The natural log–transformed values of episode duration were used as the offset.

Given that the main analysis included 26 comparisons (13 outcomes, 2 analyses per outcome), we applied a combined approach to control for multiple testing. First, two co-primary outcomes were selected (QTc and its SD during the night), for which Bonferroni correction was applied and statistical significance was set at p < 0.025. If these tests were significant, we proceeded with analysis of the remaining outcomes (secondary outcomes, n=24 tests). For these, given the hypothesis-generating nature of our study, we controlled the false discovery rate (15%) using the Benjamini–Hochberg procedure. All statistical analyses were performed using SPSS (version 28.0.1.0; IBM, Chicago, IL).

## Results

### Baseline characteristics and general description of episodes

Baseline characteristics of included and excluded participants showed no significant differences (**Table 1**).

**Table 1:** Baseline characteristics of participant by exclusion status.

Variable	Included	Excluded	P
n	23	8	
Male, n (%)	8 (34.8%)	3 (37.5%)	1.000
Age, years	36.9 ± 13.7	37.1 ± 8.3	0.968
Diabetes duration, years	20.7 ± 13	15.5 ± 7.5	0.295
Bolus insulin dose, IU	26 ± 9	21 ± 8	0.205
Basal insulin dose, IU	22 ± 10	25 ± 9	0.390
Waist, cm	86 ± 11	85 ± 8	0.901
Hip, cm	101 ± 9	102 ± 7	0.888
Weight, kg	70.4 ± 14.7	69.1 ± 10.3	0.818
Height, m	170 ± 9	166 ± 7	0.228
BMI, kg/m^2^	24.2 ± 3.4	25.1 ± 3.2	0.547
Systolic blood pressure, mmHg	124 ± 16	123 ± 7	0.783
Diastolic blood pressure, mmHg	73 ± 9	77 ± 6	0.312
HbA1c, %	8.1 [7.7, 8.6]	8.3 [7.8, 9.1]	0.642
HbA1c, mmol/mol	65 [61, 70]	67 [62, 76]	0.642
Total cholesterol, mmol/l	4.6 [4.3, 5.5]	5.4 [4.7, 5.8]	0.132
LDL cholesterol, mmol/l	2.7 [2.2, 3.4]	3.4 [3.2, 3.6]	0.074
HDL cholesterol, mmol/	1.5 [1.4, 1.8]	1.5 [1.4, 1.7]	0.520
Brain natriuretic peptide, pg/ml	14 [5, 18]	22 [5, 36]	0.249
Urinary albumin concentration, mg/l	4.6 [3.1, 7.3]	3.5 [0.5, 7.3]	0.321
TSH, mIU/l	1.1 [0.9, 1.5]	1.6 [1.1, 4.1]	0.132
Insulin pump treatment, n (%)	4 (17.4%)	2 (25.0%)	0.634
Family history of myocardial infarction, n (%)	8 (34.8%)	4 (50.0%)	0.418
Current smoking, n (%)	5 (21.7%)	3 (37.5%)	0.393
Use of beta-blocker, n (%)	4 (17.4%)	1 (12.5%)	1.000
Sensory neuropathy, n (%)	6 (26.1%)	2 (25.0%)	1.000
Autonomic neuropathy, n (%)	9 (39.1%)	3 (37.5%)	1.000
Thyroxin replacement, n (%)	1 (4.3%)	1 (12.5%)	0.389

Data are mean ± SD, median [IQR], or n (%). p values were calculated by independent samples t-test, Mann-Whitney U test, or chi-square test as appropriate.

HbA1c, glycated haemoglobin A1c; TSH, thyroid stimulating hormone.

The 23 included patients had a total of 113 hypoglycaemic and 113 control episodes. Of these episodes, 84 occurred at night and 29 during the day.

Altogether, the 226 episodes lasted 378 h and represented 1,697,205 beats. Daytime recordings lasted 227 h (hypoglycaemic episodes: 115 h, 550,940 beats; control episodes: 112 h, 519,561 beats), whereas nighttime recordings lasted 151 h (hypoglycaemic episodes: 78 h, 315,915 beats; control episodes: 73 h, 310,789 beats). Nighttime episodes were longer than daytime episodes for both hypoglycaemic and control episodes (p<0.01 for both). Mean IG levels were lower during nighttime than daytime hypoglycaemic episodes. In contrast, mean IG levels were similar during nighttime and daytime control episodes (**Table 2**).

**Table 2:** Characteristics of hypoglycaemic and control episodes.

	Hypoglycaemic episodes			Control episodes		
	Daytime	Nighttime	P	Daytime	Nighttime	P
Number of episodes	84	29	–	84	29	–
Duration of episodes, min	66 (57-75)	159 (128-192)	<0.001	63 (55-71)	150 (124-178)	<0.001
Nadir glucose, mmol/l	2.6 (2.6-2.7)	2.5 (2.3-2.6)	0.03	6.3 (5.8;6.8)	6.7 (5.8-7.7)	0.455

Estimated marginal means (95% confidence intervals) and p-values are based on linear mixed models.

### Arrhythmia analysis

**Figure 2:**
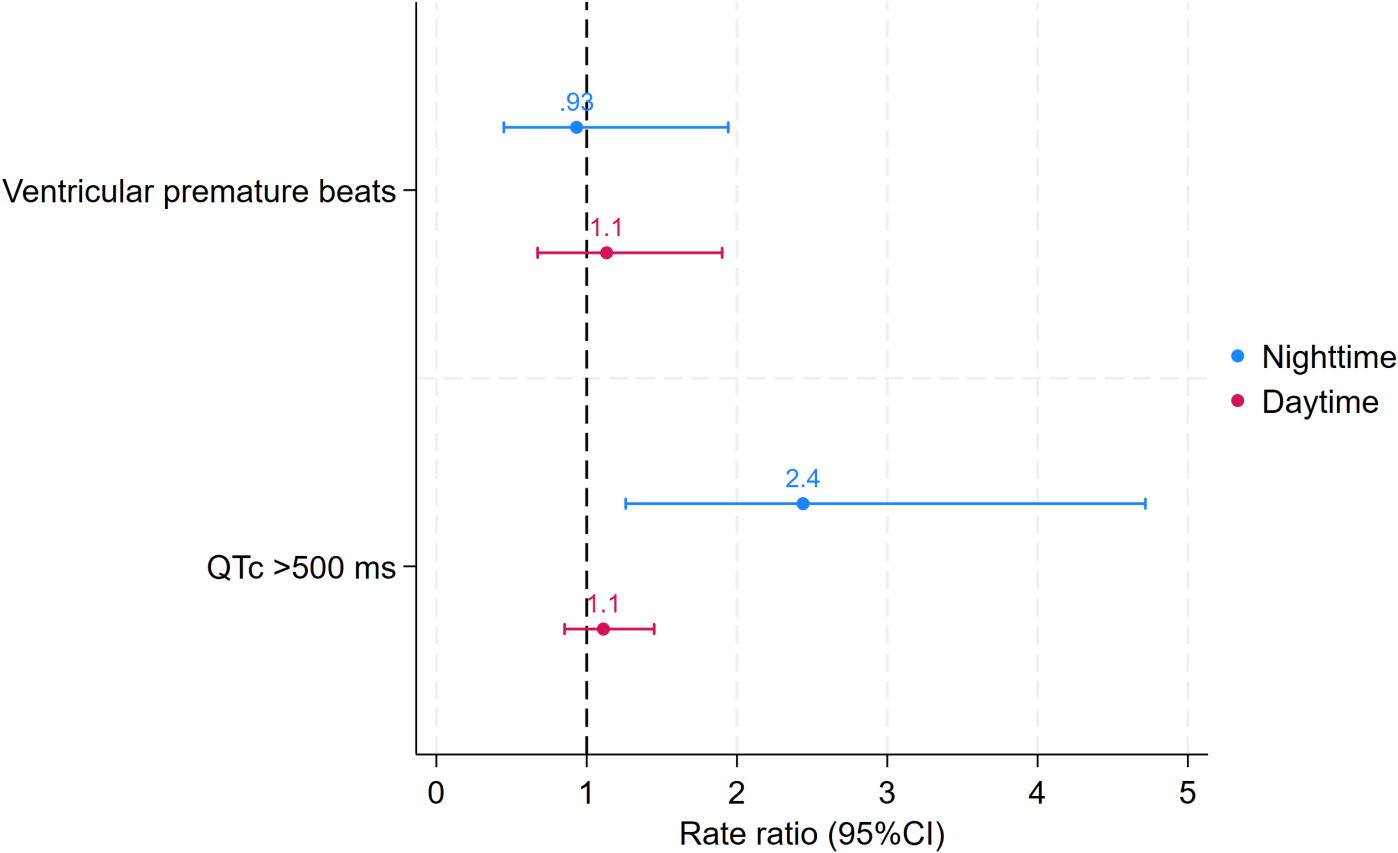
Rate ratios of ventricular premature beats and beats with prolonged QTc (>500 ms) during hypoglycaemic vs control episodes stratified by time of day. Rate ratios are based on generalised linear models. Error bars represent 95% confidence intervals (CI).

Altogether, 1,121 VPBs were detected during hypoglycaemic episodes, most occurring during the daytime (daytime: n=949, nighttime: n=172). A similar number of VPBs were detected during control episodes (daytime: n=943, nighttime: n=217). The rate of VPBs was numerically lower at night compared to the day, but no difference was found between hypoglycaemic and control episodes (daytime: 8.3, 95% CI: 3.7–18.7 vs 8.4, 95% CI: 2.4–30.1 VPBs/h; p=0.96; nighttime: 2.2, 95% CI: 0.6–8.9 vs 3.0, 95% CI: 0.9–9.4 VPBs/h; p=0.47) (**Figure 2**).

### Analysis of normal beats

HR (calculated from RR intervals) was lower at night compared to the day. However, no significant differences between hypoglycaemic and control episodes in HR were found (nighttime: 72.7, 95% CI: 68.7–76.6 vs 71.9, 95% CI: 67.9–75.8 bpm; daytime: 87.1, 95% CI: 84.8–89.5 vs 86.2, 95% CI: 83.9–88.5 bpm) (**Table 3**).

**Table 3:** Frequency of ventricular premature beats (VPBs) and lengths of ECG intervals during hypoglycaemic and control periods stratified by time of day.

Nighttime (n = 58 episodes)							
	Hypoglycaemic episodes	Control episodes	P		Hypoglycaemic episodes	Control episodes	P
	EMM (95%CI)	EMM (95%CI)			EMM (95%CI)	EMM (95%CI)	
VPBs, n/hour	2.4 (0.8-7.6)	2.6 (0.9-7.6)	0.853				
RR, ms	856 (818-895)	863 (825-902)	0.727	*SD of RR, ms*	102 (89-114)	84 (72-97)	0.01*
P, ms	112 (108-116)	113 (110-117)	0.296	*SD of P, ms*	8 (6-10)	6 (4-8)	0.018*
PQ, ms	163 (157-170)	162 (155-168)	0.384	*SD of PQ, ms*	11 (9-14)	8 (5-11)	0.014*
QRS, ms	97 (94-100)	96 (93-99)	0.123	*SD of QRS, ms*	7 (5-9)	6 (4-8)	0.065
QT, ms	404 (394-414)	402 (392-412)	0.611	*SD of QT, ms*	17 (15-19)	13 (11-15)	0.002*
QTc, ms	440 (432-448)	435 (427-443)	0.003*	*SD of QTc, ms*	21 (18-23)	16 (14-18)	<.001*
Daytime (n = 168 episodes)							
	Hypoglycaemic episodes	Control episodes	P		Hypoglycaemic episodes	Control episodes	P
	EMM (95%CI)	EMM (95%CI)			EMM (95%CI)	EMM (95%CI)	
VPBs, n/hour	7.6 (3.5-16.3)	6.7 (2.5-17.8)	0.616				
RR, ms	710 (688-733)	715 (692-737)	0.722	*SD of RR, ms*	74 (66-81)	70 (63-77)	0.335
P, ms	109 (107-111)	110 (107-112)	0.343	*SD of P, ms*	9 (8-10)	9 (8-10)	0.714
PQ, ms	149 (146-153)	150 (146-153)	0.625	*SD of PQ, ms*	14 (13-16)	15 (13-16)	0.656
QRS, ms	98 (97-100)	98 (97-100)	0.986	*SD of QRS, ms*	11 (10-12)	11 (10-12)	0.855
QT, ms	369 (364-375)	371 (366-377)	0.455	*SD of QT, ms*	15 (13-16)	14 (12-15)	0.248
QTc, ms	441 (437-446)	442 (437-446)	0.684	*SD of QTc, ms*	20 (19-21)	19 (18-20)	0.112

P values are given for the difference between hypoglycaemic and control episodes based on linear mixed models and generalised linear models. * Statistically significant differences after correction for multiple tests.

QTc intervals were calculated using Bazett’s formula.

EMM, estimated marginal mean; VPBs, ventricular premature beats; SD, standard deviation.

**Figure 3:**
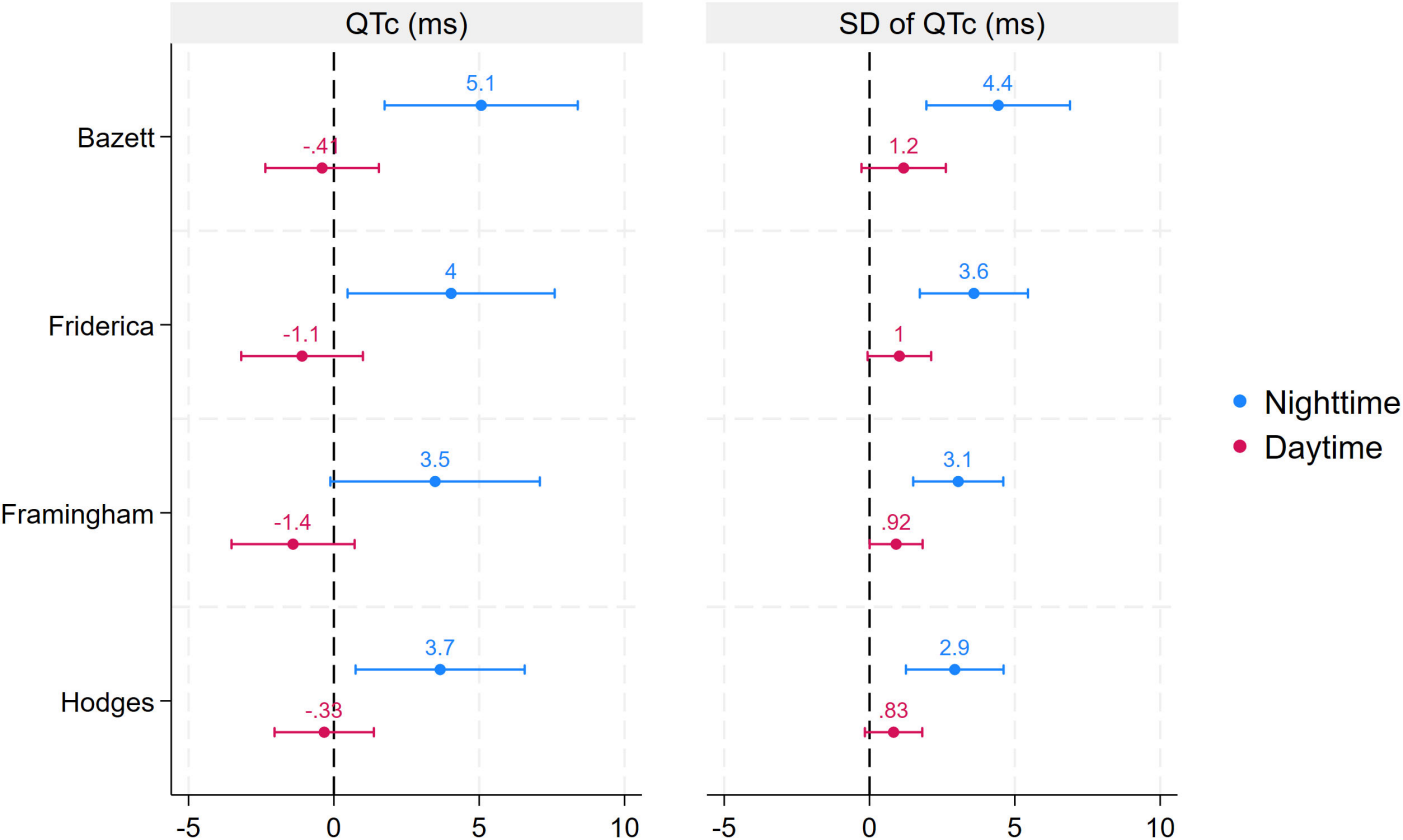
Estimated difference in the co-primary outcomes (QTc and its SD based on Bazett’s correction) as well as QTc and its SD using different methods for QT correction between hypoglycaemic and control episodes stratified by time of day. Estimated differences are based on linear mixed models. Error bars represent 95% confidence intervals.

**Figure 4:**
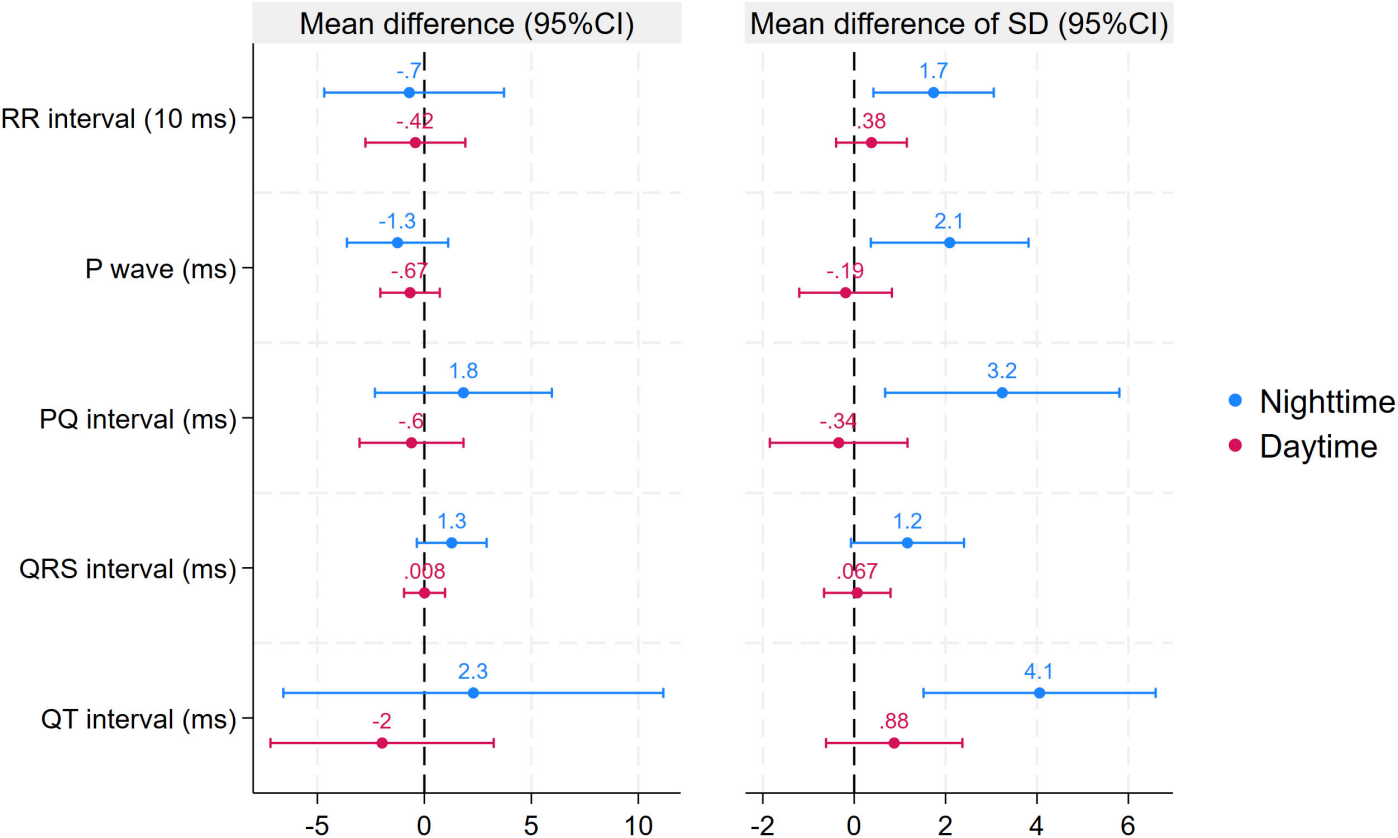
Estimated differences in the secondary outcomes between hypoglycaemic and control episodes stratified by time of day. Estimated differences are based on linear mixed models. Error bars represent 95% confidence intervals.

Next, we compared mean P-wave length and the PQ, QRS, QT, and QTc intervals during hypoglycaemic and control episodes stratified by time of day. Except for a longer nighttime QTc interval during hypoglycaemic episodes (mean difference [MD]: 5, 95% CI: 2–8 ms), no differences were found in these parameters between hypoglycaemic and control episodes (**Table 3**, **Figures 3**, **4**).

The final analysis examined the SD of the ECG parameters. At night, the SD of all parameters (except for the QRS interval) increased (RR interval MD: 17, 95% CI: 4–31; P-wave MD: 2, 95% CI: 0–4; PQ interval MD: 3, 95% CI: 1–6; QT interval MD: 4, 95% CI: 2–7; QTc interval MD: 4, 95% CI: 2–7). In contrast, no significant differences between hypoglycaemic and control episodes were found in any of these variables during the day (**Table 3**, **Figures 3**, **4**).

### Sensitivity analyses

The first set of sensitivity analyses investigated the robustness of our findings on the increase of QTc interval and its SD using different correction methods. These analyses confirmed both findings, with point estimates similar to those of the main analysis. Furthermore, the rate of normal beats with a prolonged QTc was more than doubled (rate ratio [RR]: 2.43, 95% CI: 1.26–4.72) during nighttime hypoglycaemic episodes compared to control episodes, further supporting our results for the co-primary outcomes (**Figures 2**, **3**).

**Figure 5:**
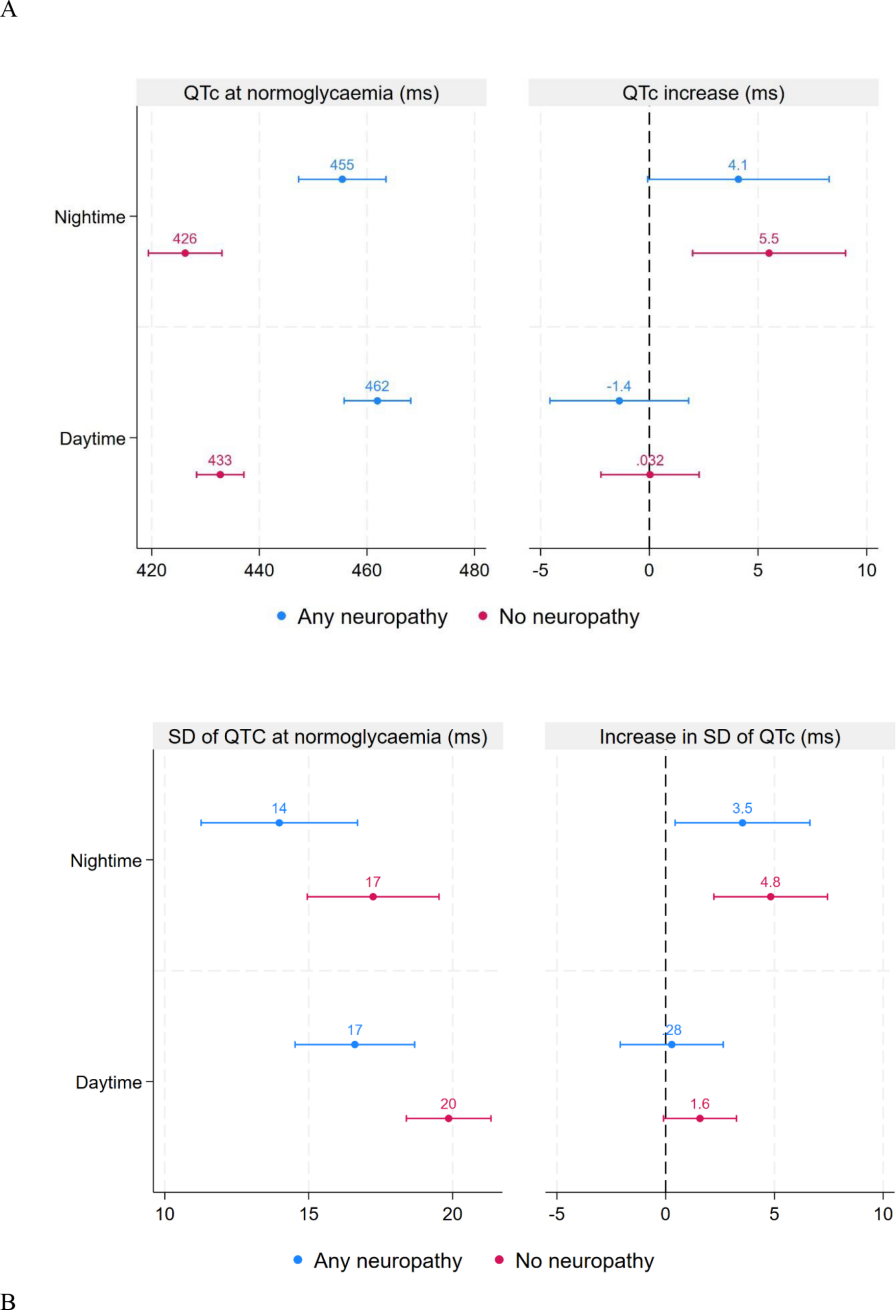
Estimated QTc **(A)** and its SD **(B)** during normoglycaemia as well as difference in QTc **(A)** and its SD **(B)** between hypoglycaemic and control episodes stratified by time of day and by neuropathy status. Estimated differences are based on linear mixed models. Error bars represent 95% confidence intervals. Any neuropathy refers to the presence of either autonomic or sensory diabetic neuropathy.

We found that the presence of neuropathy was associated with an increased QTc and decreased SD of QTc. However, there was no interaction between neuropathy and hypoglycaemia status, indicating that the changes in QTc and its SD were similar in patients with and without neuropathy: an increase during the night and no significant change during the day (**Figure 5**).

**Figure 6:**
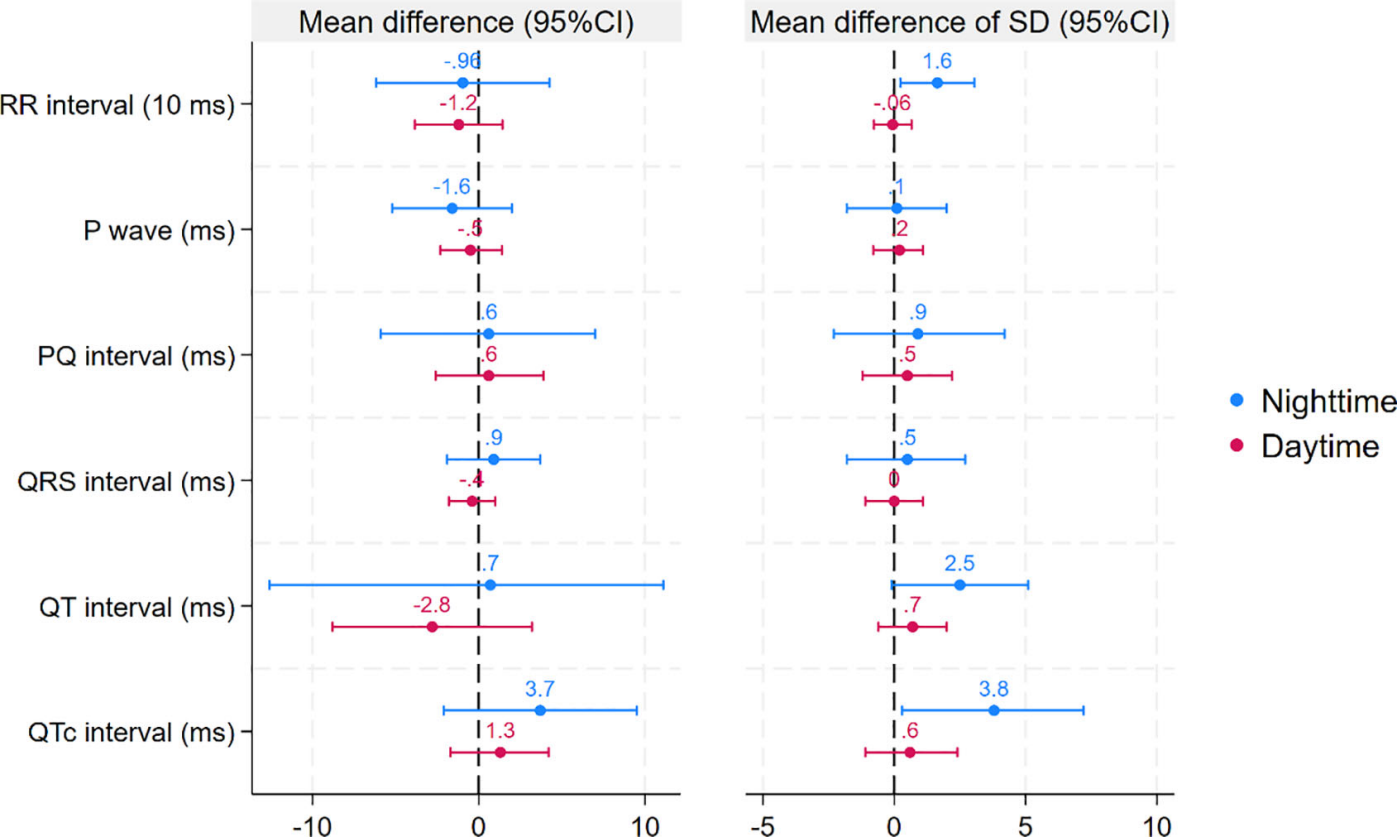
Differences in primary and secondary outcomes between nadir hypoglycaemic and control episodes stratified by time of day (sensitivity analysis). Estimated differences are based on linear mixed models. Error bars represent 95% confidence intervals.

**Figure 7:**
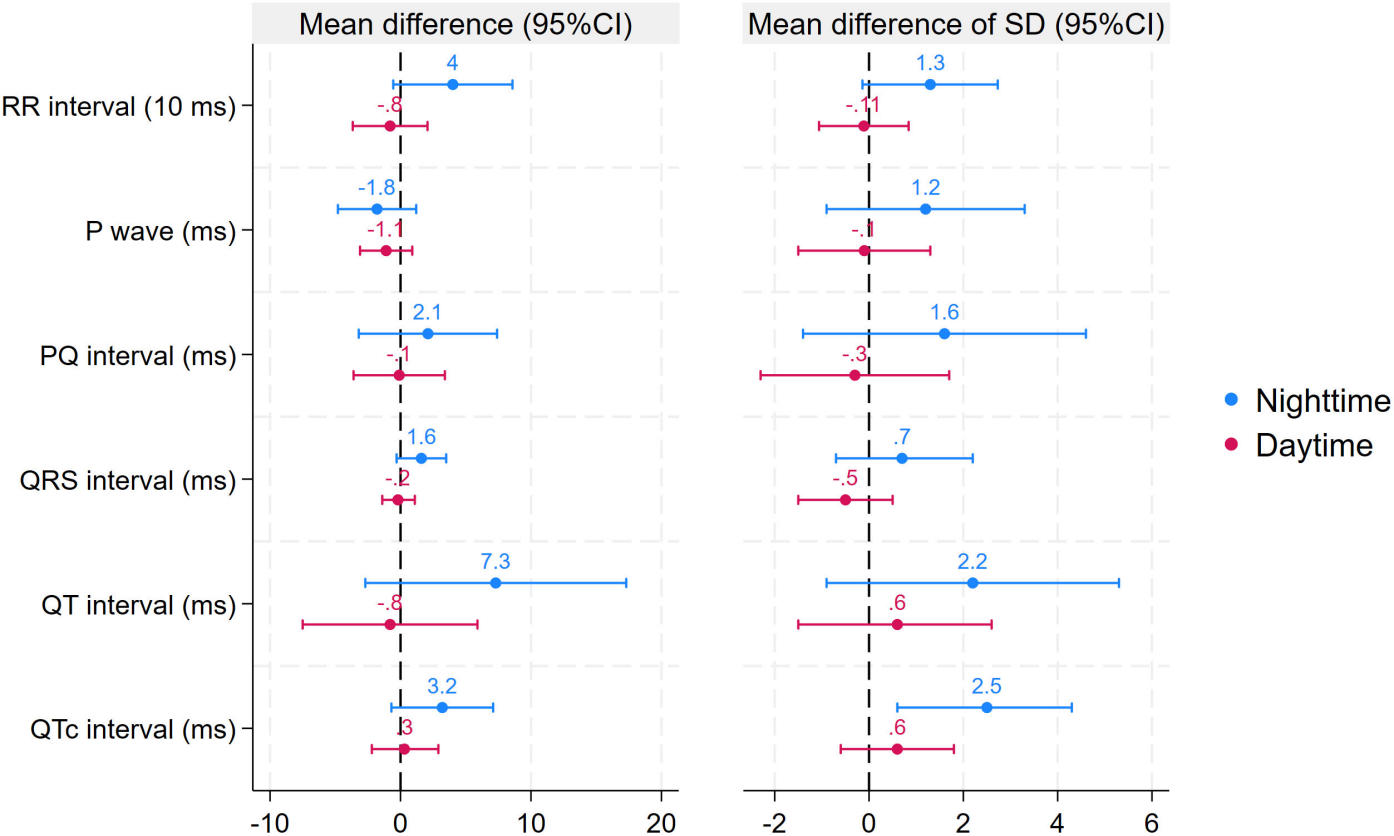
Differences in primary and secondary outcomes between hypoglycaemic and control episodes using 3 mmol/l as the cutoff for events stratified by time of day (sensitivity analysis). Estimated differences are based on linear mixed models. Error bars represent 95% confidence intervals.

The other two sensitivity analyses used different definitions for hypoglycaemic episodes. These analyses largely confirmed our main findings, demonstrating a significantly greater dispersity (SD) of QTc intervals during hypoglycaemia compared to control episodes at night. The differences in point estimates between hypoglycaemic and control episodes were similar; however, the CIs included 0 for the mean of QT and QTc, as well as for the SD of QT intervals at night. Consistent with the main analysis, no differences between hypoglycaemic and control episodes were found during the day for any of the investigated parameters (**Figures 6**, **7**).

## Discussion

In this observational study of 23 patients with type 1 diabetes free from known CVD and severe diabetes complications (1,697,205 heartbeats), we assessed the risk-related ECG changes associated with hypoglycaemic episodes. Nighttime episodes were longer than daytime episodes. Mean IG levels were lower during nighttime compared to daytime hypoglycaemic episodes, whereas they were similar during nighttime and daytime control episodes. We found no difference in the number of VPBs or in HR between hypoglycaemic and control episodes (during either nighttime or daytime). In the normal-beats analysis, we found a longer QTc interval together with an increased SD of QTc during nighttime hypoglycaemic episodes. Furthermore, we observed increases in the SD of the P-wave, PQ, QRS, and QT intervals during nighttime hypoglycaemic episodes, although the means of these parameters were similar in hypoglycaemic and control episodes. No differences in any of the investigated parameters were found between hypoglycaemic and control episodes during the day.

Although the “dead-in-bed” syndrome has been described for decades, its underlying causes remain uncertain (8, 9). Most reports hypothesize the role of hypoglycaemia-induced QTc prolongation resulting in malignant arrhythmia. Experimental studies using hypoglycaemic clamps, which report significant QTc elongations, seem to support this hypothesis (10–14). However, studies in free-living patients with diabetes found only moderate QTc increases that are unlikely to cause severe arrhythmia (15–20, 43, 44).

Most studies—similar to our findings—reported no difference, or clinically non-significant increases, in HR during hypoglycaemic versus control episodes in patients with type 1 (16, 43, 44) or type 2 diabetes (37, 45–47). Based on these findings, changes in HR are unlikely triggers of sudden cardiac death.

Previous studies using Holter ECG monitoring for <5 days (17, 20, 44) or loop recorders for 21 days (48), showed no difference in the number of VPBs between hypoglycaemic and control episodes in type 1 diabetes. In contrast, patients with type 2 diabetes and high cardiovascular risk had a higher rate of VPBs during daytime (37) or during both daytime and nighttime hypoglycaemia (49). Although some studies reported similar rates of VPBs with and without hypoglycaemia, the overall evidence suggests increased VPB rates mainly in patients with previous CVD. In contrast, healthy patients with type 1 diabetes appear to have no increase in VPB rates during hypoglycaemia.

Given the plausibility of QTc elongation as a potential cause of malignant arrhythmias, previous studies have frequently investigated QTc length. Most have reported a mean 5–10 ms prolongation in type 1 diabetes, primarily during nighttime hypoglycaemia (15, 18, 19). One study reported a daytime QTc increase (20), and one reported a non-significant change (3 ms) (16). Our findings parallel and extend these observations, demonstrating a modest increase during nighttime hypoglycaemia irrespective of the presence of diabetic neuropathy. By contrast, studies in type 2 diabetes showed no changes in QTc (37, 45, 46), although one study reported increased QTc during the day (49). Overall, the slight QTc elongation observed is an unlikely cause of malignant arrhythmias.

It is well established that a single beat with an extreme QTc duration may initiate malignant arrhythmia (21, 22). Thus, the higher end of the QTc distribution—reflected by increases in the dispersity (SD) of QTc length—could be a better indicator of arrhythmia risk than changes in the mean QTc. To the best of our knowledge, no previous studies have compared the distribution of QTc lengths during normoglycaemia versus hypoglycaemia. We found that, in addition to a slight increase in QTc, the SD of QTc also increased during nighttime hypoglycaemic compared to control episodes, irrespective of the presence of diabetic neuropathy. Consistent with this observation, we also found that the proportion of beats with QTc >500 ms more than doubled during nighttime hypoglycaemic episodes compared to control episodes. Taken together, these findings are compatible with an increased—but still very low—risk of malignant arrhythmia during nighttime hypoglycaemia. Additional congenital factors (e.g., channelopathies) or acquired conditions (e.g., diarrhoeal infection) could further increase baseline QTc, thereby exacerbating arrhythmia risk.

While we found no changes in mean P-wave, PQ, or QRS interval lengths, the SDs of P-wave length and the PQ interval increased significantly during nighttime hypoglycaemia. Although the clinical importance of these changes is not yet known, they suggest a systemic alteration in the overall cardiac electrical cycle.

Our study has strengths that must be acknowledged. First, our study benefits from a long observation period (~7 days) for each individual participant. Our data provide ample statistical power with the use of gold-standard statistical methods (mixed models) that take into account the similarity of within-person hypoglycaemic episodes. Second, our study—using strict inclusion and exclusion criteria—has excellent internal validity. Third, during data collection and cleaning, investigators were blinded to IG values. Furthermore, automatic arrhythmia analysis was supervised by the same specialist (VJH). Fourth, to control for diurnal physiological variation, we used a case–control design in which hypoglycaemic and control episodes were matched for both time of day and patient. Fifth, based on previous study results and theoretical assumptions, we selected two co-primary outcomes (QTc and a novel outcome: SD of normal-beat times) for which we corrected p values for multiple testing and conducted a set of sensitivity analyses using different QT correction methods. We also investigated the frequency of beats with prolonged QTc intervals. All these sensitivity analyses confirmed our main findings. Finally, for the secondary outcomes, we corrected for multiple comparisons by controlling the false discovery rate and also conducted sensitivity analyses using different methods to define hypoglycaemic events. These analyses further confirmed our primary results.

Our study also has limitations that must be addressed. The sensor used (Enlite) is an outdated device with lower precision compared to more recent devices. Nonetheless, the good detection rate of hypoglycaemic events reported in the literature, together with the fact that the hypoglycaemic events in our study were much longer than the reported lag time between sensor and meter glucose values, argues against significant bias related to the limited precision of the Enlite sensor. This device also requires fingerprick calibration, and the quality of the glucose measurements could bias our findings. However, all participants had long-standing diabetes and substantial experience with self-monitoring. Given the strict inclusion criteria and the monoethnic patient population, external validity is limited. Furthermore, our study was not powered to investigate the glucose cut-off at which changes in the cardiac cycle begin, and the study setting did not allow us to investigate the association between sensor glucose values as a continuous variable and cardiac cycle parameters.

## Conclusions

We found no differences between hypoglycaemic and control episodes in any of the investigated parameters during the day, suggesting that daytime hypoglycaemia has no major effect on cardiac electrophysiology and is an unlikely cause of malignant arrhythmias in otherwise healthy patients with type 1 diabetes. In contrast, QTc duration was slightly increased during nighttime hypoglycaemia. Although this change—consistent with the literature—is largely incompatible with a clinically meaningful increase in the risk of malignant arrhythmias, the significant increase in the SD of QTc indicates that some beats may reach sufficiently long durations to trigger malignant arrhythmias, even though no such events occurred in our study sample. Our findings are therefore compatible with the extremely rare occurrence of the “dead-in-bed” syndrome. Overall, our results suggest that fear of malignant arrhythmias (and the very low theoretical risk) should not be a major concern when determining individual glycaemic targets for patients with type 1 diabetes without macrovascular complications.

## Data Availability

The raw data supporting the conclusions of this article will be made available by the authors, without undue reservation.
